# The predictors of voluntary participation in pulmonary tuberculosis screening program: a study in a suburban community of southern Thailand

**DOI:** 10.3389/fpubh.2024.1360986

**Published:** 2024-04-09

**Authors:** Chanon Kongkamol, Apinya Chintrakul, Kanakorn Horsiritham, Nantaka Kiranantawat, Sitang Nirattisaikul, Jitpreedee Sungsiri, Pornchai Sathirapanya, Chutarat Sathirapanya, Koontidar Boonma, Tuck Chowwanapoonpohn, Paradon Nuiman, Jekita Supunthuchaikul, Nuttartham Chokthamangoon, Chalanthon Chintana, Trithep Suktaneekul, Chananyu Watcharanimit

**Affiliations:** ^1^Department of Family and Preventive Medicine, Faculty of Medicine, Prince of Songkla University, Songkhla, Thailand; ^2^Air Pollution and Health Effect Research Center, Prince of Songkla University, Songkhla, Thailand; ^3^Health Sciences and Clinical Research, Faculty of Medicine, Prince of Songkla University, Songkhla, Thailand; ^4^Division of Digital Innovation and Data Analytics (DIDA), Faculty of Medicine, Prince of Songkla University, Songkhla, Thailand; ^5^Department of Radiology, Faculty of Medicine, Prince of Songkla University, Songkhla, Thailand; ^6^Department of Medicine, Faculty of Medicine, Prince of Songkla University, Songkhla, Thailand

**Keywords:** tuberculosis, health, community, screening, prevention

## Abstract

**Background:**

The health belief model (HBM), baseline health condition, and sociocultural factors impact the decision to participate in a tuberculosis screening program.

**Methods:**

This cross-sectional and descriptive study was carried out among the “Kao Taew” community dwellers aged 18 years and above, who voluntarily underwent the provided pulmonary tuberculosis (PTB) screening by chest radiographs (CXRs). The level of individual HBM domain perception, attitudes toward PTB prevention, and regularity of PTB prevention practices by the participants were evaluated. The significantly associated or correlated factors such as demographic characteristics, individual HBM domain perception, and attitudes toward PTB prevention with the regularity of PTB prevention practices from the univariate analysis were further analyzed by multiple linear regression (p < 0.05) to determine the independent significant predictors of PTB prevention practices.

**Results:**

Among 311 participants comprising 65% women, 57.9% aged ≥ 65 years and 67.2% had an underlying disease. The study participants had a high level of perception of HBM domains but a low level of perception of the barrier. In addition, a high level of attitudes toward PTB prevention and a high regularity of PTB prevention practices were found. A multiple linear regression analysis revealed that the perceived benefits of PTB screening [Beta = 0.20 (0.04, 0.36) *p* = 0.016] and acquiring underlying diseases [Beta = 1.06 (0.38, 1.73), *p* = 0.002] were significant predictors for PTB prevention practices, while belief in Islam was a reverse predictor [Beta = −0.84 (−1.47, −0.21), *p* = 0.010].

**Conclusions:**

The level of perception of the individual domain of HBM, health status, and religious belief significantly predicted voluntary participation in PTB screening programs. Careful consideration by integration of the relevant health psychology, physical, and sociocultural factors is crucial for planning a health screening program.

## 1 Introduction

Pulmonary tuberculosis (PTB), a contagious pulmonary infection disease, has been a health concern globally for a long time. Stopping its spread is the highest aim of global and individual national healthcare programs. In the year 2015, the WHO endorsed an “End of tuberculosis” strategy aimed at eradicating tuberculosis by 2035 (1). Therefore, PTB control was included in Goal 3 of Sustainable Development Goals (SDGs) for control of communicable diseases, and under SDGs issue 3.3, PTB, AIDS, malaria, neglected tropical diseases, hepatitis, water-borne diseases, and other communicable diseases were the targets (2). The increasing incidence of PTB is a major health concern, and the process of its effective control is challenging. Public screening in areas posing a high PTB infection rate have been carried out in many countries. It is noteworthy that the mortality rate of patients with HIV and PTB co-infection has been decreasing, while that of non-HIV patients remained stable (3). It is possible that, besides the recent advance of anti-retrovirus drugs, regular medical check-ups and screening tests including chest radiography (CXR) can be performed. Because PTB is a usual co-infection in HIV patients, early detection and treatment can reduce the mortality rate. This finding highlights the benefit of regular health and/or CXR screening, especially among those who have an underlying immune-compromised state. While active participation in health screening programs to prevent PTB spread among the public is required, some barriers such as knowledge; socioeconomic, cultural, or religious beliefs; or conflicting psychological perceptions of the disease exist. Therefore, strengthening health education and campaigns are needed to foster public understanding and disease recognition. Understanding the perception of health and disease in potential participants is also crucial. First, evaluation of how they perceive the risk, susceptibility, and severity of the disease is mandatory (4–9). Then, removal of all possible barriers for access to the available health services, either geographic, travel, individual emotional factors, or non-rational thoughts, should be encouraged. This way of a public approach to encourage healthcare participation is based on the health belief model (HBM) (10–13).

Several global and national strategies to control the spread of PTB have been applied. Health education, campaigns, and interventions for facilitating the active participation of the public in PTB screening programs are widely implemented. Furthermore, mass screening of the public by CXRs or several laboratory techniques and providing treatments to the diagnosed PTB patients are common strategies implemented worldwide to reduce the incidence of PTB. Despite these intensified health programs, the incidence of PTB in some specific locations still increases. The barriers to access to these health programs, i.e., geographic separation or difficulty in traveling, may be one of the contributing factors. However, other factors such as individual psychosocial, economic, cultural, or religious beliefs can contribute to the non-acceptance of the well-provided health services too. While routine CXR is the simplest way for PTB screening and is widely available, the rate of active participation in screening by CXR is still lower than expected in many countries (14). The reasons for participants' reluctance to undergo CXR screening may be because clinical symptoms of PTB are less severe at the beginning and slowly progress, leading to under-recognition and perception of infection and its fatality, or it is considered a low socioeconomic stigma in some societies, causing unwillingness in people to undergo CXR screening. The discrepancy between the availability of CXR and the engagement in screening radiography by people requires further exploration.

Thailand is one of the top 14 countries worldwide with high PTB incidence. Due to the less severe pulmonary symptoms compared with other pulmonary infections and under-recognition of acquiring PTB as mentioned, it can widely spread if the infection control measures are not stringent (15). In the year 2022, there were 103,000 (143:100,000) newly diagnosed or recurrent cases of PTB reported in Thailand, among which 1,200 died of the disease (16). The successful treatment rate in Thailand during 2013–2020 was 81.5–86.3%, which was lower than the global target (90%). The reasons were as follows: 9.3% of the PTB patients died before completing the treatment, among which were patients aged > 65 years accounted for 19% of the dead and non-compliance with the treatment provided accounted for 5.4% of the dead (17). The under-recognition of acquiring PTB infection, low active participation in medical screening, and low treatment success rate together contributed to the high incidence of PTB in Thailand.

The high incidence of PTB in Thailand also impacted the situation of PTB in Songkhla, a southern province of Thailand. It was ranked as the eighth among top ten provinces with a high PTB incidence in Thailand according to the records of the Department of Disease Control, Ministry of Public Health, from October 2020 to February 2021 (18). In the first quarter of 2023, a total of 688 newly diagnosed and recurrent PTB cases were reported in Songkhla province, which accounted for 34.8% of all pulmonary infections reported in the province. Hat Yai, Meung (the study area), and Sadao districts were the top three districts reported to have a high PTB incidence in the province (16). Then, PTB screening programs for early detection and treatment, which were national policy-driven strategies for PTB control in Thailand, were implemented in this province. Routine CXR has been accepted for mass PTB screening in both community and specific settings due to its simplicity of application and high cost-effectiveness (19, 20). Although the computer-assisted CXRs for PTB screening in primary healthcare services have been suggested to increase the sensitivity of PTB detection (14), the limited healthcare resources in our country preclude its use in routine PTB screening. Furthermore, deep learning-based automated PTB detection algorithms have been introduced for PTB screening, especially in very low-incidence areas (21). Based on the high PTB incidence in Thailand, its application does not fit to the situation. While the national policy of BCG vaccination for all newborns in Thailand and other countries with high PTB incidence is applied to prevent tuberculosis infection during infancy and childhood, it loses its protective effect against PTB in adults. Cases of newly diagnosed PTB have been reported from crowded areas or areas with poor hygiene and living conditions. “Kao Taew,” which was the study community, was one of the crowded communities around the Meung district, the metropolitan city of Songkhla province. Access to health screening services has no limitations at all, but active participation in CXR screening by community residents is low. Hence, investigating the reasons for the disagreement is an urgent task for the provincial healthcare agencies and academic institutions. Moreover, immunocompromised people, i.e., adults, with an underlying health condition such as diabetes, chronic kidney disease, or cancers, which are commonly found in aged persons, people with HIV, or those with other causes of immune-compromised diseases, are highly susceptible to PTB. The growing number of older people in Thailand as per the reports [12.8 million (19.4% of Thai population)] at the end of June 2023 can affect the national incidence of PTB (22). Due to these reasons, finding of active PTB cases by mass CXR screening in the community with high PTB infection risk and providing early treatment are crucial for PTB control.

In this study, we aimed to evaluate the impacts of demographic characteristics of the screening program respondents, their attitudes, and levels of perception of individual domains of the health belief model (HBM) toward PTB prevention. Further analysis was carried out to determine the independent factors from the variables mentioned in predicting the PTB prevention practices among respondents. The evaluation of the impact of individual domains accounting for the HBM construct was specifically focused on providing insights into the powerful motivators that could generate a strong intention to participate in the screening program of the program respondents. The understanding derived from this study can be useful for designing and implementing future programs in similar settings.

### 1.1 Theoretical models

In this study, the HBM was specifically focused as a significant motivator of voluntary participation in the screening program. We believed that achieving high levels of perception in HBM domains would facilitate attitudes and adherence to recommendations of PTB prevention practice subsequently. The HBM, originally a social psychology concept, first described by Rosenstock (23), has been considered in planning health programs or services. It is a useful predictor of adoption and long-term adherence to the designed health programs, indicating the program's sustainable success. Considering the concept of the HBM, it consists of two opposite arms of encouraging and discouraging domains to adopt and adhere to health suggestions or interventions. Perceived disease susceptibility or risk, perceived disease severity, perceived benefits from adopting the suggested health program, cues to action following the recommendation, and self-efficacy are parts of the encouraging arm, whereas perceived barriers is in the discouraging arm. Aiming at high adoption and adherence to the program from the participants, promoting a high level of perception of risk of acquiring a disease, susceptibility, and disease severity should be stressed. Meanwhile, to lessen or remove the perception of barriers to engaging in a health program, detailed cues to action should be meticulously introduced to the participants. Then, self-efficacy and self-confidence for conducting the advised health practices will be formed in the program participants. It was suggested that the HBM alone or enhanced by self-efficacy, a high level of knowledge, or attitudes toward disease prevention were important motivators for high adoption and adherence to a health program (4–6, 24–27).

In addition to HBM, the internal health locus of control (IHLC) can enhance self-efficacy for proper self-management of one's health. A previous study suggested that facilitating the self-efficacy of program participants through enhancing the perception of HBM and strengthening belief in IHLC concurrently were useful for achieving the expected outcomes of a health program (28). Furthermore, another study stressed that self-efficacy was possibly more powerful than the HBM or other health psychology concepts in predicting sustainable adherence to health advice or programs by the program participants (29).

In conclusion, this study mainly integrated the HBM with IHLC constructs to advise the required health practice of the program to participants. We expected the formation of self-efficacy in the participants finally so that they could deliberately decide to join and follow the program activities with confidence.

### 1.2 Terms and definitions and concepts used in this study

The terms and definitions used in the study included the following:

(a). *Health Belief Model (HBM)* is a psychological construct describing the fundamental factors that influence an individual's decision to participate in a health program, follow health advice, or accept a suggested treatment or disease prevention. It consists of the domains that evaluate an individual's perception of disease vulnerability or risk, disease severity, benefits of adherence to the health advice or services, barriers to access to the services or to follow the health advice provided, and cues to action.
(b). *Self-efficacy* indicates the level of an individual's self-confidence in managing one's health condition competently.
(c). *PTB prevention practices* refers to the expected health behaviors or practices implemented to protect a person from contracting PTB.

## 2 Methods

### 2.1 Study setting and design

The current study was conducted in “Kho Taew,” a subdistrict under the governance of Meung district, the metropolitan city of Songkhla province, southern Thailand. Covering an area of 28.4 km^2^, it is located 14 km north of the Meung district where every governmental service, including healthcare, is available. The communication between the study area and the metropolitan city is convenient on the road. There are two primary health care units (PHCUs) in the study area. Kho Taew was selected as the study site due to the reported high incidence of PTB. The Songkhla Provincial Public Health Office reported that Meung district, including Kho Taew, ranked the second highest PTB incidence in the province. This study aimed to understand the perception of PTB health burden and the motivators or barriers of the community people to participate in a provided PTB screening program in such a high-risk PTB area, particularly when considering that they resided near the regular health-service centers of the province that were accessible without any difficulties. The study design used in this study was a cross-sectional, exploratory, and descriptive design.

### 2.2 Sampling methods

In our study, we invited all community dwellers aged 18 years and above, who were a high-risk group for contracting PTB, to voluntarily participate in the PTB screening program by conventional CXRs and enrolled them in this study. We understood that this method might cause selection bias, but we extensively explored the significant motivators or barriers to participation in the screening program, either personal demographic, socioeconomic, religious belief, or levels of perception of individual domains of the HBM.

### 2.3 Study method

We employed a cross-sectional and descriptive design. The primary data including demographic characteristics, level of perception of an individual domain of HBM toward PTB prevention, attitudes, and regularity of practice of PTB prevention were collected by personal interviews of the program participants.

#### 2.3.1 Study tools

The tools for data collection were the questionnaires developed by researchers to evaluate the perception, attitudes, and regularity of PTB prevention practices in the community. The questionnaires had passed the content validity and reliability tests before employment as shown by the index of item objective congruence (IOC) and Cronbach's coefficient, respectively, addressed below.

There were three designed questionnaires evaluating (a) the levels of perception of individual domains of the health belief model, (b) attitudes toward PTB prevention, and (c) the regularity of PTB prevention practices performed by the community members. All the questionnaires were tested for content validity by three experts in PTB prevention and treatment [index of item objective congruence (IOC) > 0.5]. The content reliability assessment values by Cronbach's alpha test were 0.87 and 0.90 for (a) and (b), respectively.

(a). Perception of HBM domains (six interview domains, with the score ranging from 5 to 25 points/domain). The answers to each interview question according to HBM domains were classified into five levels: strongly agree, agree, uncertain, disagree, and strongly disagree (5 points/level).
(b). Attitudes toward PTB prevention (ten interview items, with the score ranging from 1 to 5 points/item, total scores 10–50 points). A 5-point Likert scale consisting of strongly agree, agree, uncertain, disagree, and strongly disagree was used (1 point/level).
(c). Regularity of PTB prevention practices performed (five interview items, with the score ranging from 1 to 5 points/item, total score 5–25 points). In addition, five levels of regularity of PTB prevention behaviors included always, frequently, sometimes, rarely, and never were applied (1 point/level).

Then, the scores were categorized with Best's method. The individual HBM domain perception was classified as follows: low (5–11.66), moderate (11.67–18.33), and high (18.34–25.00). The total scores of attitudes toward PTB prevention were classified as follows: strongly disagree (10–17.9), disagree (18.0–25.9), uncertain (26.0–33.9), agree (34.0–41.9), and strongly agree (42.0–50.0). For the regularity of PTB prevention practices, it was classified into three levels as follows: low (5–11.66), moderate (11.67–18.33), and high regularity of practice (18.34–25.00).

#### 2.3.2 Data collection

After the ethical approval and informed written consent from the participants were obtained, the interviews for data collection were carried out by a group of well-trained fifth-year medical students as a part of their study in community medicine. Then, the CXRs for PTB screening were performed. We collected general demographic characteristics data of the study participants, the levels of perception of individual HBM domains related to PTB prevention, and attitudes and regularity of PTB prevention practices. A standard screening CXR was performed by a mobile radiological imaging machine (Fujifilm, model FDR Smart X^R^). The findings from chest images were confirmed by two independent radiologists in our institution. If there was a disagreement between the initial reports, the consensus for the final diagnosis was obtained by discussion with a third independent and clinically blind radiologist. If an abnormal CXR was suggestive of PTB, the patient would be transferred for diagnosis confirmation and treatment accordingly at Songkhla Provincial Hospital. The interviews and PTB screening radiographs were performed during 9–11 May 2023.

#### 2.3.3 Data analysis

Descriptive statistics were used to describe general demographic characteristics. The Wilcoxon-rank sum test was used to test the significant associations between general demographic characteristics and regularity of PTB prevention practices. The correlations between the level of perception in individual domains of HBM, attitudes, and regularity of PTB prevention practices were analyzed by Spearman's correlation (*p* < 0.05). The variables showing significant associations or correlations were further analyzed by multiple linear regression analysis to determine the significantly independent predictors for carrying out PTB prevention practices regularly (*p* < 0.05).

The justification for selecting this study method was based on the requirement to assess the real and current HBM perceptions, attitudes, and regularity of PTB prevention practices in the community. Hence, the descriptive and exploratory analyses were considered suitable to respond to the study objectives.

### 2.4 Study population and sampling technique

#### 2.4.1 Study population

The study population consisted of dwellers of “Kho Taew” subdistrict of Meung district, Songkhla, who were aged 18 years or above and voluntarily participated in the PTB screening by CXRs done in the community. This population was at risk for PTB contraction due to high incidence of PTB in their community.

#### 2.4.2 Sampling technique

Our sampling technique was non-randomized and inclusive. We enrolled all community dwellers who voluntarily participated in the CXR screening program. This approach was chosen because these groups are generally considered to be at higher risk for PTB as mentioned before. The rationale of using non-randomization was specifically to gain comprehensive insights into their perceptions of the HBM and its prediction of the decision to participate in the PTB screening program.

### 2.5 Determination of sampling size

The sample size for our study was calculated based on the requirements for statistical power, which resulted in a total of 262 participants. This figure was derived by using a calculation formula for a standard sample size for the multiple linear regression analysis. The specific parameters used in this formula included an effect size of 0.05 and a power of 0.95. These values were chosen to ensure that the study had adequate power to detect statistical significance and was also feasible within the constraints of the study setting and population.

The choice of an effect size of 0.05 was based on conventional standards in epidemiological research, which aimed to detect small-to-moderate effects in community-based studies. The power of 0.95 was selected to provide a high probability of correctly rejecting the null hypothesis (i.e., detecting a true effect) if it indeed exists. This high level of power reduces the risk of Type II errors, ensuring that the study findings are robust and reliable.

In accounting for the response rate, our approach in the study was to invite all eligible individuals in the community. We then monitored the actual number of participants who voluntarily participated in the study and received the PTB screening radiographs. This method ensured that we reached the required sample size of 262 participants.

### 2.6 Variable measurements

The dependent variable in this study was the regularity of PTB prevention practice. We derived the outcomes from the questionnaire evaluating the regularity of PTB prevention. The questions used to evaluate this dependent variable are shown in Table 4, reporting the regularity of PTB prevention practices.

The independent variables were the community dwellers' demographic characteristics, attitudes of PTB prevention, and level of perception of individual domains of the HBM. These variables were obtained by a personal interview with the program participants. The levels of perception of individual domains of the HBM and attitudes were evaluated through designed and validated questions in the related questionnaires, as indicated (Tables 2, 3).

The scoring and stratification of the scores obtained in different levels were described in the study tools.

### 2.7 Ethical consideration

We confirmed that we strictly followed the regulations of the 1964 Declaration of Helsinki and related standard ethical guidelines in conducting this study. Consents for participation and publication of the study were obtained from all participants. The participants' personal or identifiable information were completely anonymous.

In addition, the study protocol was reviewed and approved by the ethics committee of the Faculty of Medicine, Prince of Songkla University, an institutional ethic review board (EC. code. 66–177–9-2).

## 3 Results

### 3.1 Population, livelihoods, and healthcare service of the study area

During the study period, “Koa Taew” comprised 2,463 households with 11,519 people, including 8,176 aged 18 years or above according to the subdistrict civil registration. The original and majority of the people here followed Islam (Thai Muslim). Agriculture, e.g., rice fields and rubber plantations, followed by raising livestock were the main livelihoods (30). There were two PHCUs in this area, each of which was headed by one professional nurse and two–three assistants. Most of the Kho Taew people usually visit one of the PHCUs for initial healthcare and medical treatment. In case of complicated medical conditions, the patients are transferred to Songkhla Provincial Hospital for specific investigations and treatments. Based on the 2022 annual report of the Songkhla Provincial Public Health Office, Meung district including “Kao Taew” people ranked second for high PTB incidence in the province (16).

### 3.2 Study population demographic characteristics

A total of 317 community dwellers who voluntarily participated in PTB screening programs were enrolled in this study. Two participants were excluded as they had been diagnosed with PTB and were under treatment, and four participants did not meet the inclusion criteria. Subsequently, 311 of 8,176 (3.8%) people were included in the interviews for data collection before the CXR screening was done. Although the calculated adequate sample size was 262 people, we included all the program participants because of their voluntary participation, and informed consents were obtained for study enrollment. They consisted of 202 (65%) women and 109 (35%) men, among whom 57.9% were aged 65 years or older and only 4.5% were uneducated. Inadequate monthly earnings were reported by 60.5% of the study participants during the interview. Two-thirds (67.2%) of them had one or more underlying health conditions, e.g., essential hypertension, diabetes mellitus, dyslipidemia, chronic airway disease, and HIV infection. Twenty-six participants were current smokers (8.2%). Only six participants (1.9%) were living in the same house with a person currently diagnosed with PTB (Table 1). The high frequency of older people in the community is currently a real situation found around Thailand. The longevity of people due to advanced medical care and being free from employment are the reasons of the high proportion of the older community members involved. However, they frequently have one or more underlying health conditions, leading to an immune-compromised state. This situation elevates the risk of PTB contraction among them.

**Table 1:** General demographic characteristics of the study participants.

**Variable**		***n* (%)**	
**Sex**			
	Male	109	(35)
	Female	202	(65)
**Age (yrs.)**			
	< 65	131	(42.1)
	>65	180	(57.9)
**Body mass index (Kg/m** ^2^ **)**			
	Underweight (< 18.0)	18	(5.8)
	Normal (18.5–22.9)	108	(34.7)
	Overweight (23.0–24.9)	81	(26.0)
	Obesity (25.0–29.9)	75	(24.1)
	Severe obesity (>30)	29	(9.3)
**Education**			
	Uneducated	14	(4.5)
	Primary school	182	(58.5)
	Junior high school	36	(11.6)
	Senior high school/vocational certificate	30	(9.7)
	Higher vocational certificate/diploma	11	(3.5)
	Bachelor's degree	36	(11.6)
	Others	2	(0.6)
**Religion**			
	Buddhism	146	(46.9)
	Islam	165	(53.1)
**Monthly income**			
	Balance with the expense	23	(39.5)
	In debt	7	(2.3)
	Less than the expense	181	(58.2)
**Previous COVID-19 infection**			
	Yes	140	(45.1)
	No	171	(54.9)
**Underlying disease(s)**			
	Yes	209	(67.2)
	No	102	(32.8)
**Living in the same house with a patient having PTB**			
	Yes	6	(1.93)
	No	305	(98.07)
**Smoking**			
	Never or ex-smoker	285	(91.8)
	Current smoking	26	(8.2)

### 3.3 Levels of perception of individual HBM domains toward PTB prevention

The levels of perception of individual domains of the HBM toward PTB prevention practices were high, except for perceived barriers. This finding implied that the people of the Kao Taew subdistrict were aware of the risk of PTB contraction or susceptibility, severity of PTB infection, and benefits from participation in the PTB screening program. Therefore, they were willing to participate in the PTB screening program provided in their community and perform the PTB prevention practices (Table 2). The provision of the screening program readily available in their community possibly removed the perception of barriers of access to the program. In summary, when the community people had a clear understanding and recognition concerning the harmfulness of PTB combined with removing or lessening the barriers, they would feel convenient and willing to participate in the screening program.

**Table 2:** Scores and levels of perception of individual HBM domains.

**Domains of HBM**	Median score^*^(Q1, Q3)	**Level^#^**
Perceived susceptibility	20 (19, 23)	High
Perceived severity	20 (19, 23)	High
Perceived benefits	21 (20, 23)	High
Perceived barriers	10 (7, 14)	Low
Self-efficacy	21 (20, 24)	High
Cues to action	22 (20, 24)	High

^*^Score ranges from 5 to 25 for individual domains. ^#^Low (5–11.66), moderate (11.67–18.33), and high (18.34–25.00).

### 3.4 Levels of attitudes toward PTB prevention

The overall median score of attitudes toward PTB prevention in the community was 34 (27.5, 40.0), which was graded as agreement with PTB prevention practices. The finding could be assumed that the people of the Kho Taew subdistrict had positive attitudes toward PTB prevention (Table 3). The positive direction of attitudes toward PTB prevention was a good baseline factor for applying PTB control strategies.

**Table 3:** Scores and levels of attitudes toward PTB prevention.

**Interviewed items**	Median score^*^(Q1, Q3)	**Level**
I feel disgusted by patients who have PTB.	2 (2, 4)	Disagree
I will not buy items from a shop owner who has had PTB, even if they have been treated and cured.	2 (2, 4)	Disagree
I will not participate in community activities if I know that there are individuals who have previously had tuberculosis	3 (2, 4)	Uncertain
I feel uneasy to be in close contact with patients who have contracted PTB.	4 (3, 5)	Agree
Patients with PTB should stay at home and not engage in social activities.	4 (2, 5)	Agree
I avoid visiting the families of people who have contracted PTB.	3 (2, 4)	Uncertain
I will not allow my children to play with children from the families with a history of PTB contraction	4 (3, 5)	Agree
Restaurants should have limited zones for their service to tuberculosis patients.	4 (3, 5)	Agree
Individuals who contract PTB usually have poor household hygiene and living conditions	4 (2, 4)	Agree
It is advisable to separate tourist attractions, activities, and public areas specifically for PTB patients.	4 (3, 5)	Agree
Total score of attitudes toward PTB prevention in the community#	34 (27.5, 40)	Agree

^*^Score ranges from 1 to 5 for each item. ^#^Total score ranged from 10 to 50. Stratified levels as follows: strongly disagree (10–17.9), disagree (18.0–25.9), uncertain (26.0–33.9), agree (34.0–41.9), and strongly agree (42.0–50.0).

### 3.5 Adherence to PTB prevention practices

The overall median (Q1, Q3) score of regularity of following PTB prevention practices among the Kho Taew subdistrict people in this study was 21 (18.0, 23.0), which was graded as “high regularity” of practice. This finding indicated that the study participants had practiced the expected PTB prevention behaviors much regularly. Moreover, it was also a good baseline factor, particularly when combined with a high level of attitudes (Table 3), for strengthening the PTB prevention behaviors among the study population (Table 4).

**Table 4:** Scores evaluating regularity of PTB prevention practices.

**Items**	Median score^*^(Q1, Q3)	**Level**
I wear a facemask when exposed to unhealthy environments	5 (4, 5)	Always practice
I wash my hands before and after contamination with others' secretions	4 (4, 5)	Frequent practice
I remove phlegm and infected wastes by burying or pouring it into the toilet and then watering it with water or toilet cleaner	4 (3, 5)	Frequent practice
I open the doors and windows of the bedroom for good ventilation	4 (4, 5)	Frequent practice
I use my plates, spoons, cups, and other items separately from the others'.	4 (3, 5)	Frequent practice
Total score of PTB prevention practices#	21 (18, 23)	High regularity

^*^Score ranges from 1 to 5 for each item. ^#^Total score ranged from 5 to 25. Stratified levels as: low (5–11.66), moderate (11.67–18.33), and high regularity of practice (18.34–25.00).

### 3.6 Associations and correlations between demographic characteristics, levels of perception of individual HBM domains, and levels of attitudes toward PTB prevention, and regularity of doing PTB prevention practices

Gender (female) (*p* = 0.024), religion (Buddhism) (*p* = 0.004), and having an underlying disease (*p* = 0.004) were significantly associated with regularity of following PTB prevention practices, whereas educational levels, income, and staying in the same house with a person infected by PTB were not (Table 5).

**Table 5:** Associations and correlations between general demographic characteristics and regularity of PTB prevention practices.

**Variable**		**PTB prevention practices scores Median (Q1, Q3)**	***P*-value**
**Sex**			
	Male	20 (18, 21)	0.024^a*^
	Female	21 (19, 23)	
**Age (years)**			
	< 65	21 (18, 23)	0.878^a^
	>65	21 (18, 23)	
**BMI (Kg/m** ^2^ **)**			
	Underweight (< 18)	18.5 (17.25, 30)	0.122^b^
	Normal (18.5–22.9)	21 (19, 22.25)	
	Overweight (23–24.9)	20 (18, 23)	
	Obesity (25–29.9)	21 (18, 23)	
	Severe obesity (>30)	21 (19, 23)	
**Education**			
	Uneducated	20 (18, 22.75)	0.088^b^
	Primary school	21 (19, 23)	
	Junior high school	21 (19, 23)	
	Senior high school/vocational certificate	19.5 (15, 21.75)	
	Higher vocational certificate/diploma	23 (18.5, 23.5)	
	Bachelor	18.5 (17, 22.25)	
	Others	19 (18, 20)	
**Religion**			
	Buddhism	21 (19, 23)	0.004^a*^
	Islam	20 (18, 22)	
**Income**			
	Enough	21 (18, 23)	0.622^a^
	In debt	21 (18, 22)	
	Not enough	23 (21.5, 24.5)	
**Underlying disease**			
	Yes	21 (19, 23.25)	0.004^a*^
	No	20 (17, 20)	
**Living in the same house with a patient acquiring PTB**			
	Yes	21 (18, 23)	0.275^a^
	No	20 (18, 23)	
**Smoking**			
	Not smoking	21 (18, 23)	0.181^a^
	Current smoking	19.5 (17.25, 21)	

^a^Wilcoxon rank sum test, ^b^Spearman's rank correlation, ^*^p < 0.05.

For individual domains of HBM, levels of perceived susceptibility (rho 0.13, *p* = 0.025), perceived benefits (rho 0.19, *p* = 0.001), self-efficacy (rho 0.21, *p* = 0.002), and cues to action (rho 0.11, *p* = 0.046) had significant correlations with regularity of following PTB prevention practices, while the level of attitudes toward PTB prevention in the community did not (Table 6).

**Table 6:** Correlations between the perception of individual HBM domains and attitudes toward PTB prevention in the community and regularity of PTB prevention practices.

**Variable**	**rho**	***P*-value**
Perceived susceptibility	0.13	0.025^*^
Perceived severity	0.06	0.310
Perceived benefits	0.19	0.001^*^
Perceived barriers	−0.05	0.310
Self-efficacy	0.21	0.002^*^
Cues to action	0.11	0.046^*^
Attitudes toward PTB prevention	0.05	0.050

Spearman's rank correlation, ^*^p < 0.05.

By the linear regression analysis, we found that having an underlying disease and perceived benefit domain of the HBM significantly predicted the adoption of PTB prevention practices or also screening programs according to this study, while belief in Islam had an inverse significant prediction (Table 7).

**Table 7:** Independent predictors of practicing PTB prevention practices from demographic characteristic data and perception of individual HBM domains.

**Variable**	**Simple linear regression**		**Multiple linear regression**				
	**b**	***P*** **-value**	**Beta**	**95% CI**	**Std error**	***t*** **-value**	***P*** **-value**
Sex (Female)	0.61	0.169	0.56	−0.10 to 1.21	0.334	1.671	0.096
Religion (Islam)	−1.00	0.036^*^	−0.84	−1.47 to −0.21	0.321	−2.609	0.010^*^
Underlying disease (Yes)	1.28	0.003^*^	1.06	0.38 to 1.73	0.341	3.092	0.002^*^
Perceived susceptibility	0.16	0.707	–	–	–	–	–
Perceived benefits	0.33	0.128	0.20	0.04 to 0.36	0.082	2.413	0.016^*^
Self-efficacy	0.27	0.118	–	–	–	–	–
Cues to action	0.21	0.957	0.07	−0.09 to 0.22	0.080	0.818	0.414

Multiple R^2^: 0.1161; Adjusted R^2^: 0.1014. ^*^p < 0.05.

## 4 Discussion

Our study in Kho Taew subdistrict enrolled more female (65%) than male participants. This difference was possibly because female participants were usually concerned about their health more than males participants (31). In addition, household economic factors required males and younger people to work far from their homes for livelihoods. Therefore, more female participants and older people (aged ≥ 65 years) (57.9%) were left in the community and enrollable for this study. Another explanation could be that the older people were more likely to feel concern about their high vulnerability and actively participated in this PTB screening program. We found from the baseline participants' characteristics that female participants, those who believed in Buddhism, and who had underlying disease were significantly associated with high regularity of following PTB prevention practices, whereas economic status and living with a family member who had PTB were not (Table 5). Therefore, gender, previous illness, and religious beliefs significantly influenced the regularity of PTB prevention practices and possibly including voluntary participation in the PTB screening program in this study subsequently.

The study participants had a high level of attitudes toward the prevention of PTB spreading and high regularity of practicing PTB prevention based on the median (Q1, Q3) of total scores obtained (Tables 3, 4). In addition, the perception of individual HBM domains among the community dwellers was high, except for perceived barriers (Table 2). The combination of these findings could imply that the community dwellers realized the benefits of and perceived no significant barriers to follow PTB prevention advice or practices. We believed that, besides the high attitudes toward PTB prevention and high perception of advantages of PTB prevention following individual domains of the HBM, the health information received from usual public health education could promote their understanding regarding PTB prevention. Hence, the Kho Taew dwellers were willing to adopt the PTB prevention advice, including the CXR screening provided. Moreover, we believed that the higher percentage of educated people in the community influenced the decision of community dwellers to undergo the CXR screening as well. Our finding was supported by those studies assessing the impact of health knowledge, attitudes, and the health belief model on disease screening (10, 26, 27, 32, 33) Most of the current studies evaluated the impact of the HBM on the decision of people to undergo breast, cervical, lung, or colonic cancer screening (34–38). No studies discussing PTB screening in the community are available. A qualitative study examining the HBM domains among African immigrants who declined to participate in the provided hepatitis B screening program revealed that lack of HBV knowledge and awareness and cultural challenges related to healthcare access or preventive care, fear, and social stigma were significant barriers (39). The fear of adverse effects from COVID-19 vaccination is also an example of the barrier of acceptance of the vaccine despite public awareness of the disease fatality (40–42). The available evidence signified enhancing the perception of risk of acquiring a disease and disease severity through comprehensive health education alongside providing cues to action and weakening the barriers (40, 42).

When the significantly associated demographic characteristic variables and correlated HBM domains with regularity of performing PTB prevention practices were further analyzed to determine the significantly independent predictors of adopting PTB prevention practices (Table 7), we found that having an underlying disease, perceived benefits of PTB prevention or screening, and belief in Islam (inverse prediction) were the significant independent predictors. Several previous studies confirmed the significant influence of mostly perception of benefits and barriers of HBM domains and level of understanding or knowledge on the people's decision to participate in cancer screening or health education programs (7–10, 32). Moreover, health educations focusing on encouraging the domains of the HBM, reducing all possible barriers to access to healthcare service, and understanding how to practice disease prevention could promote self-efficacy in practicing self-care finally (11–13, 33, 43–46). For religious beliefs, the results showed a significantly inverse prediction of believing in Islam for the regularity of PTB prevention practices in this study. We believe that it is possibly explained by misunderstanding of the people, for example, the belief that getting a disease is God's will and unavoidable or a kind of fate. In addition, this way of thought follows the external health locus of controls (EHLC) concept in which the unopposed external influencers, i.e., God or bad fortune, influence the individual's self-efficacy in managing one's own health. People posing this kind of thought will lose their self-confidence in managing their own matters, including health. Earlier studies found that a high level of belief in the EHLC also negatively influenced an individual's health practice (47–51). Changing one's belief in the EHLC, particularly God- or bad fortune-attributed control, to the IHLC and strengthening one's self-efficacy should be done by demonstrating objective evidence of health outcomes after following the provided health advice (52). Importantly, health educational programs for specific population groups such as being female, married, and Muslim to provide cues to action and to form positive health attitudes without conflicts against cultural or religious beliefs were encouraged (40, 42, 53).

We specifically determined the significantly independent predictors from demographic characteristics, levels of perception in individual domains of the HBM, and attitudes that predicted the regularity of PTB prevention practices among the study community dwellers. Otherwise, we also explored the impact of socioeconomic, cultural, and religious belief on the practices. We believed that understanding of these factors would provide useful insights into their influence on the decisions of the community dwellers. In addition, comprehensive and integrated consideration of these influencing factors is essential for program success. We stressed on the significant impact of the HBM as a powerful driver in facilitating the decision of the community people to participate in the health program. Therefore, detailed assessment of the perception of individual domains of the HBM before planning and applying a health program is crucial.

## 5 Strength and limitation of the study

By focusing on a specific community located near the metropolitan city of Songkhla province, where access to healthcare service is not a problem, we found that strengthening the perception of risk and severity of PTB to individual health and reducing barriers of access based on the HBM construct could clear the unmet practice for PTB screening in the community. Additionally, we further suggested that, by forming self-efficacy, self-care ability could be developed among the community people.

This study has some limitations. It had a small sample size and was performed in a specific study location that would limit its generalizability. Primary data were obtained from the interviews in which response bias from the participants could be involved. Moreover, the knowledge about PTB provided before data collection might modify the real perception, attitudes, and practices. Finally, the requirement of the community people to undergo CXR screening for PTB possibly deviated the responses causing bias as well.

## 6 Conclusion

There is a gap between the national policy-driven PTB control and the response from the people in the community in this study. Multiple factors including demographics, religion belief, and perception of disease according to the HBM concept can affect the decision to accept the screening advice. Comprehensive evaluation of these factors is mandatory before careful planning of a health program for the community can be initiated. Enhancing the encouraging domains of HBM concept and lessening the influence of barriers of access to healthcare service are essential for the success of the program. In this regards, suitable health education provided to the community people is needed.

Since different social context affect the health belief, decision, and practice of the people, we suggest that expanding the sample size and study setting to cover people having various socioeconomic and health statuses, cultures, and religious beliefs will benefit from designing a policy-driven community health program in the future.

## Data availability statement

The original contributions presented in the study are included in the article/supplementary material, further inquiries can be directed to the corresponding author.

## Ethics statement

The studies involving humans were approved by the Ethics Committee of the Faculty of Medicine, Prince of Songkla University (EC code no. 66–177–9-2). The studies were conducted in accordance with the local legislation and institutional requirements. The participants provided their written informed consent to participate in this study.

## Author contributions

CK: Conceptualization, Data curation, Formal analysis, Methodology, Software, Supervision, Validation, Writing – original draft, Writing – review & editing. AC: Data curation, Formal analysis, Validation, Writing – review & editing. KH: Data curation, Formal analysis, Validation, Writing – review & editing. NK: Data curation, Investigation, Validation, Writing – review & editing. SN: Data curation, Investigation, Validation, Writing – review & editing. JS: Data curation, Investigation, Validation, Writing – review & editing. PS: Validation, Writing – original draft, Writing – review & editing. CS: Conceptualization, Data curation, Formal analysis, Methodology, Supervision, Validation, Visualization, Writing – review & editing. KB: Data curation, Validation, Writing – original draft. TC: Data curation, Validation, Writing – original draft. PN: Data curation, Validation, Writing – original draft. JS: Data curation, Validation, Writing – original draft. NC: Data curation, Validation, Writing – original draft. CC: Data curation, Validation, Writing – original draft. TS: Data curation, Validation, Writing – original draft. CW: Data curation, Validation, Writing – original draft.

## References

[1] Division of Tuberculosis Control Ministry of Public Health Thailand. National Plan for Pulmonary Diseases Control 2017–2021. (2021). Available online at: https://www.oic.go.th/FILEWEB/CABINFOCENTER28/DRAWER068/GENERAL/DATA0001/00001041.PDF (accessed February 17, 2024).

[2] WHO. The Global Health Observatory: Sustainable Development Goals Target 3.3: Communicable Diseases. (2024). Available online at: https://www.who.int/data/gho/data/themes/topics/sdg-target-3_3-communicable-diseases (accessed Feburary 9, 2024).

[3] WHO Global Tuberculosis Program. Global Tuberculosis Report 1997–2022. (2024). Available online at: https://www.who.int/teams/global-tuberculosis-programme/tb-reports (accessed February 9, 2024).

[4] Lau J Lim TZ Jianlin Wong G Tan KK. The health belief model and colorectal cancer screening in the general population: a systematic review. Prev Med Rep. (2020) 20:101223. 10.1016/j.pmedr.2020.101223 33088680 PMC7567954

[5] AlJunidel R Alaqel M AlQahtani SH AlOgaiel AM ALJammaz F Alshammari S. Using the health belief model to predict the uptake of mammographic screening among saudi women. Cureus. (2020) 12:e11121. 10.7759/cureus.11121 33133789 PMC7586377

[6] Gemeda EY Kare BB Negera DG Bona LG Derese BD Akale NB. Prevalence and predictor of cervical cancer screening service uptake among women aged 25 years and above in Sidama Zone, Southern Ethiopia, using health belief model. Cancer Control. (2020) 27:1073274820954460. 10.1177/1073274820954460 32951445 PMC7791476

[7] Belay AS Asmare WN Kassie A. Cervical cancer screening utilization and its predictors among women in bench Sheko Zone, Southwest Ethiopia: using health belief model. BMC Cancer. (2023) 23:472. 10.1186/s12885-023-10927-x 37221482 PMC10204309

[8] AlShamlan NA AlOmar RS AlAbdulKader AM AlGhamdi FA Aldakheel AA Al Shehri SA. Beliefs and utilization of cervical cancer screening by female health care workers in Saudi Arabia using the health belief model: a nationwide study. Int J Women Health. (2023) 15:1245–59. 10.2147/IJWH.S415924 37576181 PMC10417788

[9] AL-Hammadi FA Al-Tahri F Al-Ali A. Limited understanding of pap smear testing among women, a barrier to cervical cancer screening in the United Arab Emirates. Asian Pac J Cancer Prev. (2017) 18:3379–87. 10.22034/APJCP.2017.18.12.3379 29286607 PMC5980898

[10] Ma C. An investigation of factors influencing self-care behaviors in young and middle-aged adults with hypertension based on a health belief model. Heart Lung. (2018) 47:136–41. 10.1016/j.hrtlng.2017.12.001 29395265

[11] Hu Y Liu H Wu J Fang G. Factors influencing self-care behaviours of patients with type 2 diabetes in China based on the health belief model: a cross-sectional study. BMJ Open. (2022) 12:e044369. 10.1136/bmjopen-2020-044369 35953256 PMC9379478

[12] Habibi H Sedighi B Jahani Y Hasani M Iranpour A. Self-care practices and related factors in patients with multiple sclerosis (MS) based on the health belief model. J Caring Sci. (2021) 10:77–83. 10.34172/jcs.2021.015 34222116 PMC8242295

[13] Damghanian M Mahmoodzadeh H Khakbazan Z Khorsand B Motaharinezhad M. Self-care behaviors in high-risk women for breast cancer: a randomized clinical trial using health belief model education. J Educ Health Promot. (2020) 9:265. 10.4103/jehp.jehp_76_20 33282970 PMC7709765

[14] Moodley N Velen K Saimen A Zakhura N Churchyard G Charalambous S. Digital chest radiography enhances screening efficiency for pulmonary tuberculosis in primary health clinics in South Africa. Clin Infect Dis. (2020) 74:1650–8. 10.1093/cid/ciab644 34313729

[15] WHO Global Tuberculosis Program. Global Tuberculosis Report 2020. (2020). Available online at: https://apps.who.int/iris/bitstream/handle/10665/336069/9789240013131-eng.pdf (accessed February 10, 2024).

[16] Songkhla Provincial Public Health Office. Newsletter of the Songkhla Provincial Public Health Center. (2023). Available online at: https://www.skho.moph.go.th/web/news.php?id=1016 (accessed August 19, 2023).

[17] Division of Tuberculosis Control Ministry of Public Health Thailand. Clinical Practice Guideline Tuberculosis Preventive Treatment 2023. (2023). Available online at: https:tbthailand.org (accessed February 10, 2024).

[18] Department of Disease Control Ministry of Public Health Thailand. Situation and surveillance of tuberculosis in Thailand. (2023). https://www.tbthailand.org/ (Accessed August 19, 2023).

[19] Wahedi K Biddle L Bozorgmehr K. Cost-effectiveness of targeted screening for active pulmonary tuberculosis among asylum-seekers: a modelling study with screening data from a German federal state (2002-2015). PLoS ONE. (2020) 15:e0241852. 10.1371/journal.pone.0241852 33151980 PMC7644037

[20] Velayutham B Jayabal L Watson B Jagadeesan S Angamuthu D Rebecca P. Tuberculosis screening in household contacts of pulmonary tuberculosis patients in an urban setting. PLoS ONE. (2020) 15:e0240594. 10.1371/journal.pone.0240594 33057399 PMC7561118

[21] Lee JH Park S Hwang EJ Goo JM Lee WY Lee S. Deep learning-based automated detection algorithm for active pulmonary tuberculosis on chest radiographs: diagnostic performance in systematic screening of asymptomatic individuals. Eur Radiol. (2021) 31:1069–80. 10.1007/s00330-020-07219-4 32857202

[22] Department of Older Persons Ministry of Social Development and Human Security. The Report of Older Persons in Thailand. (2023). Available online at: https://www.dop.go.th/th/statistics_side?content=1&sub=2 (accessed September 20, 2023).

[23] Rosenstock IM. Why people use health services. Milbank Mem Fund Q. (1966) 44:94–127. 10.2307/3348967 5967464

[24] Dehghani-Tafti A Mazloomy Mahmoodabad SS Morowatisharifabad MA Afkhami Ardakani M Rezaeipandari H Lotfi MH. Determinants of self-care in diabetic patients based on health belief model. Glob J Health Sci. (2015) 7:33–42. 10.5539/gjhs.v7n5p33 26156902 PMC4803867

[25] Baghianimoghadam MH Shogafard G Sanati HR Baghianimoghadam B Mazloomy SS Askarshahi M. Application of the health belief model in promotion of self-care in heart failure patients. Acta Med Iran. (2013) 51:52–8. 23456585

[26] Ruiz-González I Fernández-Alcántara M Guardia-Archilla T Rodríguez-Morales S Molina A Casares D. Long-term effects of an intensive-practical diabetes education program on HbA1c and self-care. Appl Nurs Res. (2016) 31:13–8. 10.1016/j.apnr.2015.12.008 27397812

[27] Usman IM Chama N Aigbogun EO Kabanyoro A Kasozi KI Usman CO. Knowledge, attitude, and practice toward cervical cancer screening among female university students in Ishaka Western Uganda. Int J Womens Health. (2023) 15:611–20. 10.2147/IJWH.S404845 37082233 PMC10112480

[28] Náfrádi L Nakamoto K Schulz PJ. Is patient empowerment the key to promote adherence? A systematic review of the relationship between self-efficacy, health locus of control and medication adherence. PLoS ONE. (2017) 12:e0186458. 10.1371/journal.pone.0186458 29040335 PMC5645121

[29] Karimy M Araban M Zareban I Taher M Abedi A. Determinants of adherence to self-care behavior among women with type 2 diabetes: an explanation based on health belief model. Med J Islam Repub Iran. (2016) 30:368. 27493912 PMC4972051

[30] Website for Halal Tourism and Behaviors of Tourists in Five Southern Border Provinces of Thailand Linked to the Area of Indonesia-Malaysia-Thailand Growth Triangle (IMT-GT). (2023). Available online at: https://www.me-fi.com/tourismdb/halaltourism-imt-gt/subdistric_detail.php?subid=2 (accessed August 10, 2023).

[31] Szymczyk I Wojtyna E Lukas W Kepa J Pawlikowska T. How does gender influence the recognition of cardiovascular risk and adherence to self-care recommendations? A study in Polish primary care. BMC Fam Pract. (2013) 14:165. 10.1186/1471-2296-14-165 24175983 PMC3818445

[32] Melkamu L Berhe R Handebo S. Does patients' perception affect self-care practices? The perspective of health belief model diabetes. Metab Syndr Obes. (2021) 14:2145–54. 10.2147/DMSO.S306752 34012280 PMC8128344

[33] Naeemi L Daniali SS Hassanzadeh A Rahimi M. The effect of educational intervention on self-care behavior in hypertensive older people: applying the health belief model. J Educ Health Promot. (2022) 11:406. 10.4103/jehp.jehp_1800_21 36824083 PMC9942131

[34] Al-Ani A Hammouri M Sultan H Al-Huneidy L Mansour A Al-Hussaini M. Factors affecting cervical screening using the health belief model during the last decade: a systematic review and meta-analysis. Psychooncology. (2024) 33:e6275. 10.1002/pon.6275 38282232

[35] Bak M Chin CL Chin J. Use of health belief model-based deep learning to understand public health beliefs in breast cancer screening from social media before and during the COVID-19 pandemic. AMIA Annu Symp Proc. (2024) 2023:280–288. 38222395 PMC10785880

[36] Vigneshwaran E Goruntla N Bommireddy BR Mantargi MJS Mopuri B Thammisetty DP. Prevalence and predictors of cervical cancer screening among HIV-positive women in rural western Uganda: insights from the health-belief model. BMC Cancer. (2023) 23:1216. 10.1186/s12885-023-11683-8 38066496 PMC10709934

[37] Khazaei S Salmani F Moodi M. Evaluation of health belief model-based educational intervention on colorectal cancer screening behavior at South Khorasan, Iran. J Educ Health Promot. (2022) 11:52. 10.4103/jehp.jehp_279_21 35372600 PMC8974927

[38] Ramezankhani A Akbari ME Soori H Ghobadi K Hosseini F. The role of the health belief model in explaining why symptomatic Iranian women hesitate to seek early screening for breast cancer: a qualitative study. J Cancer Educ. (2023) 38:1577–83. 10.1007/s13187-023-02302-y 37115346

[39] Wang M Qureshi A Johnson N Mansalay A Muhr A Abatemarco DJ. A health belief model examination of factors related to hepatitis B screening among African immigrants in Philadelphia. J Racial Ethn Health Disparities. (2023). 10.1007/s40615-023-01841-w 37878235

[40] Patwary MM Disha AS Hasan M Bardhan M Hasan M Tuhi FI. Integrating health belief model and theory of planned behavior to assess COVID-19 vaccine acceptance among urban slum people in Bangladesh. PLoS ONE. (2023) 18:e0290412. 10.1371/journal.pone.0290412 38117841 PMC10732453

[41] Khazir Z Kouhpeikar H Rahaei Z Zareipour M Dashti S Gholamian M. The predictors of the intention to receive COVID-19 vaccine using the health belief model and theory of planned behavior in South Khorasan province. J Educ Health Promot. (2023) 12:405. 10.4103/jehp.jehp_1480_22 38333152 PMC10852185

[42] Nasiratu I Pencille LB Khuzwayo N Aboagye RG Tarkang EE. Predictors of COVID-19 vaccine uptake among persons aged 18 years and above in Ga North Municipality, Ghana using the Health Belief Model: a community-based cross-sectional study. PLoS ONE. (2023) 18:e0293350. 10.1371/journal.pone.0293350 37934776 PMC10629641

[43] Sadeghi R Tol A Moradi A Baikpour M Hossaini M. The impacts of a health belief model-based educational program on adopting self-care behaviors in pemphigus vulgaris patients. J Educ Health Promot. (2015) 4:105. 10.4103/2277-9531.171819 27462647 PMC4946272

[44] Thahirabanuibrahim F Logaraj M. The effect of the health belief model education for cervical cancer prevention, screening promotion among rural women in Chengalpattu district, Tamil Nadu (HBMECC). J Educ Health Promot. (2023) 12:166. 10.4103/jehp.jehp_1133_22 37404911 PMC10317253

[45] He L Gao S Tao S Li W Du J Ji Y. Factors associated with colonoscopy compliance based on Health Belief Model in a community-based colorectal cancer screening program Shanghai, China. Int Q Commun Health Educ. (2020) 41:25–33. 10.1177/0272684X19897356 31876256

[46] Gholampour Y Jaderipour A Khani Jeihooni A Kashfi SM Afzali Harsini P. The effect of educational intervention based on health belief model and social support on the rate of participation of individuals in performing fecal occult blood test for colorectal cancer screening. Asian Pac J Cancer Prev. (2018) 19:2777–87. 10.22034/APJCP.2018.19.10.2777 30360606 PMC6291048

[47] Afsahi F Kachooei M. Relationship between hypertension with irrational health beliefs and health locus of control. J Educ Health Promot. (2020) 9:110. 10.4103/jehp.jehp_650_19 32642466 PMC7325753

[48] Tahmasebi R Noroozi A. Is health locus of control a modifying factor in the health belief model for prediction of breast self-examination? Asian Pac J Cancer Prev. (2016) 17:2229–33. 10.7314/APJCP.2016.17.4.2229 27221923

[49] Abredari H Bolourchifard F Rassouli M Nasiri N Taher M Abedi A. Health locus of control and self-care behaviors in diabetic foot patients. Med J Islam Repub Iran. (2015) 29:283. 26913246 PMC4764266

[50] Bennett BL Goldstein CM Gathright EC Hughes JW Latner JD. Internal health locus of control predicts willingness to track health behaviors online and with smartphone applications. Psychol Health Med. (2017) 22:1224–9. 10.1080/13548506.2017.1317354 28415852 PMC5873305

[51] Lim JW Gonzalez P Wang-Letzkus MF Ashing-Giwa KT. Understanding the cultural health belief model influencing health behaviors and health-related quality of life between Latina and Asian-American breast cancer survivors. Support Care Cancer. (2009) 17:1137–47. 10.1007/s00520-008-0547-5 19050938

[52] Demir E Yildirim E. The effect of religious belief on the attitudes of pregnant's toward the fetal health. J Relig Health. (2019) 58:2313–23. 10.1007/s10943-019-00818-6 30972610

[53] Ampofo AG Adumatta AD Owusu E Awuviry-Newton K A. cross-sectional study of barriers to cervical cancer screening uptake in Ghana: an application of the health belief model. PLoS ONE. (2020) 15:e0231459. 10.1371/journal.pone.0231459 32352983 PMC7192489

